# First person – Youwen Zhang

**DOI:** 10.1242/dmm.049278

**Published:** 2021-10-18

## Abstract

First Person is a series of interviews with the first authors of a selection of papers published in Disease Models & Mechanisms, helping early-career researchers promote themselves alongside their papers. Youwen Zhang is first author on ‘ Propensity to endoplasmic reticulum stress in deer mouse fibroblasts predicts skin inflammation and body weight gain’, published in DMM. Youwen is a PhD student in the lab of Hippokratis Kiaris at the University of South Carolina, Columbia, SC, USA, investigating the underlying pathogenic mechanisms associated with disruption of energy and protein homeostasis in organisms.


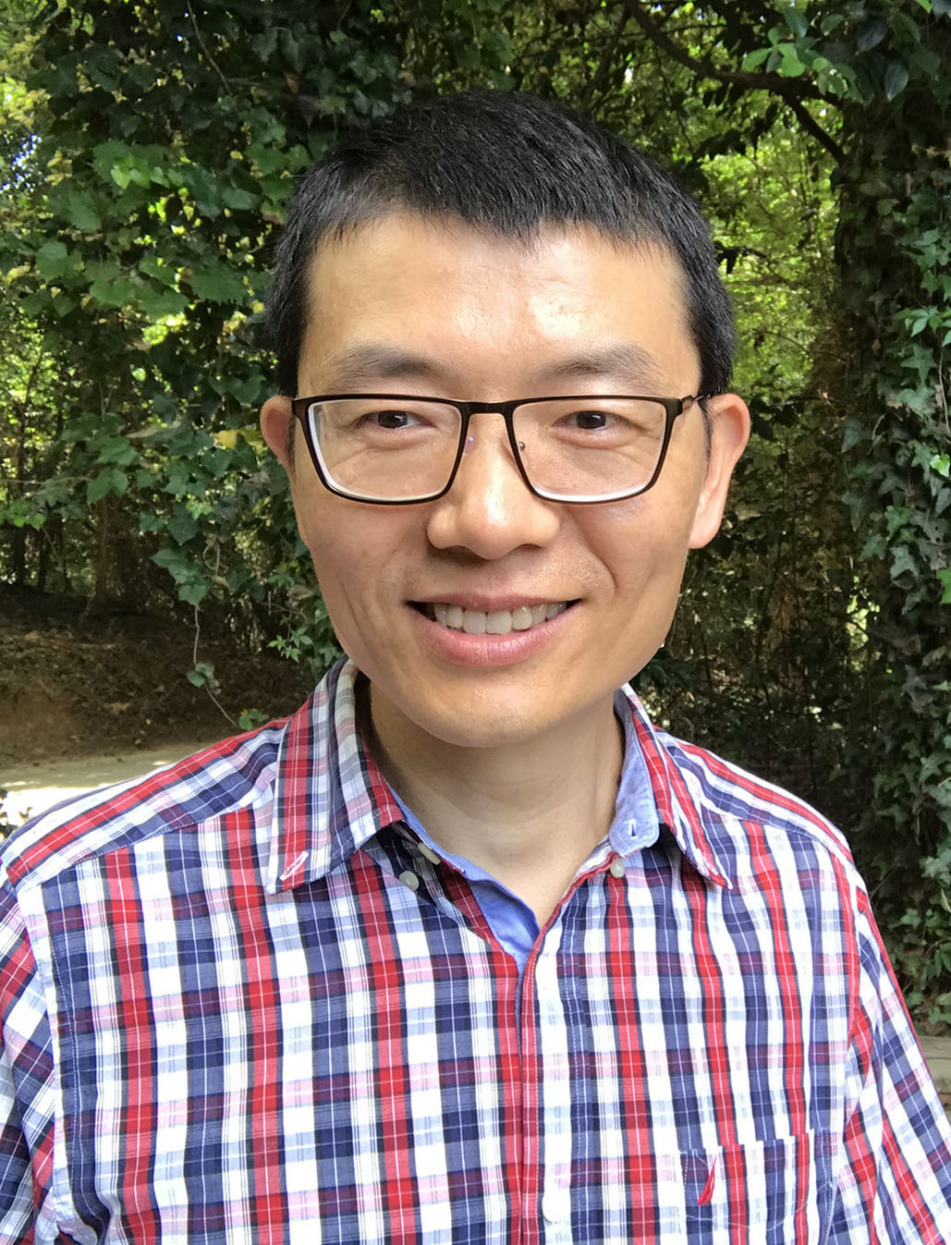


**Youwen Zhang**

**How would you explain the main findings of your paper to non-scientific family and friends?**

The endoplasmic reticulum (ER) is an organelle found in most eukaryotic cells and plays an important role in protein folding and lipid metabolism. Nutrient excess triggers ER stress and unfolded protein response (UPR), a signalling network initiated to facilitate protein folding and restore normal ER function. In this research, we show that the intensity of UPR in fibroblasts isolated early in life predicts the extent of body weight gain after high-fat diet (HFD) administration using outbred deer mice as a model. Contrary to those with intense UPR, animals with moderate UPR in fibroblasts and therefore displaying compromised stress resolution did not gain body weight but developed inflammation, especially in the skin, after HFD administration.

“Obesity has reached epidemic proportions worldwide, placing a huge health and economic burden on society.”

**What are the potential implications of these results for your field of research?**

Obesity has reached epidemic proportions worldwide, placing a huge health and economic burden on society. Over-nutrition disrupts energy balance in the body, but different people show different tolerability of nutritional disturbance. By using deer mice, a genetically diverse animal model that can better reflect human population compared to inbred laboratory mice, we have identified potential underlying causes of the different responses to over-nutrition in different individuals. Also, the prediction of body weight gain by the intensity of UPR in fibroblasts isolated early in life provides a possible tool for the early prevention and intervention of pathogenesis of obesity and metabolic syndrome.

**What are the main advantages and drawbacks of the model system you have used as it relates to the disease you are investigating?**

The deer mice we used in this study were from the Peromyscus Genetic Stock Center at the University of South Carolina, where the outbred colonies of various *Peromyscus* species have been maintained via careful breeding for more than 30 years. As an outbred animal model, they are genetically diverse and better reflect the variations seen in human population than inbred laboratory mice. Contrary to laboratory mice that, depending on the specific strain, may show a significant increase in body weight after HFD consumption, deer mice in our study showed a wide range of body weight change, which provides a valuable model for the exploration of variations that may confer the susceptibility to metabolic pathogenesis induced by nutritional disturbance. Nevertheless, there are some drawbacks for this animal model. The genomic and analytical tools are limited for deer mice, which restricts some genetic manipulations and molecular analyses of cells and tissues. Also, the wide variance among animals may reduce the statistical power in certain tests and analysis. Therefore, a larger sample size and/or alternative statistical approaches may be needed for data acquisition and processing.

**Figure:**
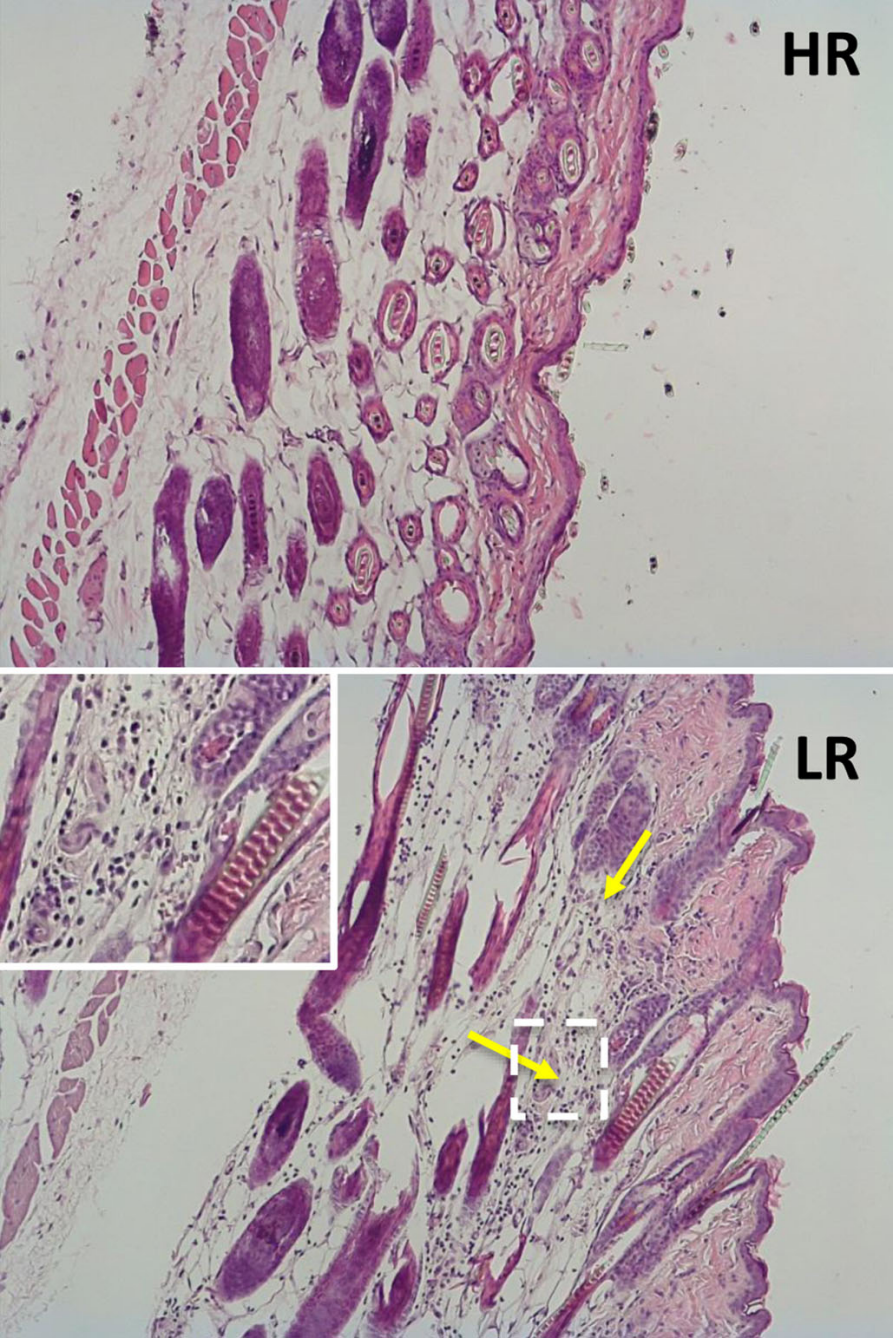
**Deer mouse skin tissue sections from high UPR responders (HR) and low UPR responders (LR).** Despite the same HFD consumption, minimal inflammation was seen in the skin of HR (top) whereas a high degree of inflammation was shown in the skin of LR (bottom). Yellow arrows indicate areas with high immune cell infiltration; the area in the dashed line box is also shown in the upper-left corner at a higher magnification.

**What has surprised you the most while conducting your research?**

Molecular chaperones within the ER play an important role in the folding of newly synthesized proteins and the maintenance of ER homeostasis. The levels of ER chaperone expression induced by ER stress inducers have long been seen as major indicators of the severity of ER stress in cells. No research has yet examined the connection between the levels of chaperone expression and the capacity of ER stress resolution in cells. When comparing the expression levels of three major ER chaperones – BiP, GRP94 and calnexin – with the accumulation of unfolded protein aggregates in cells, we were amazed to find a significant negative correlation between them, which suggests that a higher chaperone induction under the same stress conditions is associated with more efficient ER stress resolution. This discovery also helps shed light on the understanding why the individuals with high UPR showed better tolerance to HFD while those with low UPR were sick and developed inflammation after HFD consumption.

**Describe what you think is the most significant challenge impacting your research at this time and how will this be addressed over the next 10 years?**

The availability of genetic and molecular tools for deer mice is still limited currently. For example, it is hard to conduct morphological studies in tissue- and subcellular-level studies in cells without antibodies produced specifically against deer mice. I believe this issue will be addressed when the specificity and importance of deer mice as an animal model for human diseases attract more and more researchers' attention.

“A concerted effort and continuing financial and non-financial support are urgently required to ensure sustainable progress in scientific research and improve the professional lives of early-career scientists.”

**What changes do you think could improve the professional lives of early-career scientists?**

The uncertainty of academic career and the insufficient financial support are very challenging for early-career scientists, especially for those working on basic scientific research. The ongoing pandemic may stimulate governments, funding agencies and the public to rethink the importance of fundamental science conducted by researchers. A concerted effort and continuing financial and non-financial support are urgently required to ensure sustainable progress in scientific research and improve the professional lives of early-career scientists.

**What's next for you?**

We are currently working on whole-genome single-nucleotide polymorphism analysis in deer mice with high or low UPR in fibroblasts during ER stress. This may help us to unravel the genetic fundamentals bringing the variations in the UPR among different individuals.
